# AirLab: a cloud-based platform to manage and share antibody-based single-cell research

**DOI:** 10.1186/s13059-016-1006-0

**Published:** 2016-06-29

**Authors:** Raúl Catena, Alaz Özcan, Andrea Jacobs, Stephane Chevrier, Bernd Bodenmiller

**Affiliations:** Institute of Molecular Life Sciences, University of Zürich, Winterthurerstrasse 190, Building/Room: Y11-J-82, CH-8057 Zurich, Switzerland; Department of Biology, Biology Master’s Program, ETH, Zürich, Switzerland

## Abstract

Single-cell analysis technologies are essential tools in research and clinical diagnostics. These methods include flow cytometry, mass cytometry, and other microfluidics-based technologies. Most laboratories that employ these methods maintain large repositories of antibodies. These ever-growing collections of antibodies, their multiple conjugates, and the large amounts of data generated in assays using specific antibodies and conditions makes a dedicated software solution necessary. We have developed AirLab, a cloud-based tool with web and mobile interfaces, for the organization of these data. AirLab streamlines the processes of antibody purchase, organization, and storage, antibody panel creation, results logging, and antibody validation data sharing and distribution. Furthermore, AirLab enables inventory of other laboratory stocks, such as primers or clinical samples, through user-controlled customization. Thus, AirLab is a mobile-powered and flexible tool that harnesses the capabilities of mobile tools and cloud-based technology to facilitate inventory and sharing of antibody and sample collections and associated validation data.

**Electronic supplementary material:**

The online version of this article (doi:10.1186/s13059-016-1006-0) contains supplementary material, which is available to authorized users.

## Introduction

Technologies such as multicolor flow cytometry and mass cytometry enable researchers to analyze multiple molecules, such as proteins and their modifications, in thousands of single cells in a rapid manner [1]. These two methodologies are critical in the field of single-cell biology. We recently developed imaging mass cytometry [2], which allows multiparametric analysis in tissue sections, facilitating single-cell biology studies in the context of each cell’s neighborhood and larger morphological features of tissues [2]. Other high-throughput technologies for single-cell analysis are single-cell sequencing [3] and a multitude of microfluidic approaches [4]. Experimental procedures for these single-cell analysis technologies are backed by large inventories of antibodies or DNA probes. Most laboratories and clinics that routinely use such techniques also house large reagent stocks, for example, antibodies labeled in multiple ways with fluorophores, metals, or other molecular tags. Currently, inventory of these stocks and data associated with each sample are managed using commercial database software packages such as FileMaker, Access, or Excel [5] or tools designed more specifically for research laboratories, such as Quartzy [6] (https://www.quartzy.com) or Labguru [6] (http://www.labguru.com). Quartzy is focused on the organization of inventory and ordering whereas Labguru is specifically designed for laboratory project management. Topic-specific databases for biomedical research, such as Swissprot (http://web.expasy.org/docs/swiss-prot_guideline.html), miRBase [7], and PhosphoPep [8], have also been developed in the past two decades. Databases aimed to aid antibody-aided research also exist, including the Antibody Registry (http://www.antibodyregistry.org), antibodypedia [9], and CiteAb [10]. These are useful resources in which practical data about titration, applications, background noise, and pitfalls are described. None of these tools, however, were developed integrally to handle antibody collections, to design and manage antibody panels, to enable sharing of antibody validation data, or to seamlessly connect the user with available databases.

**Fig. 1:**
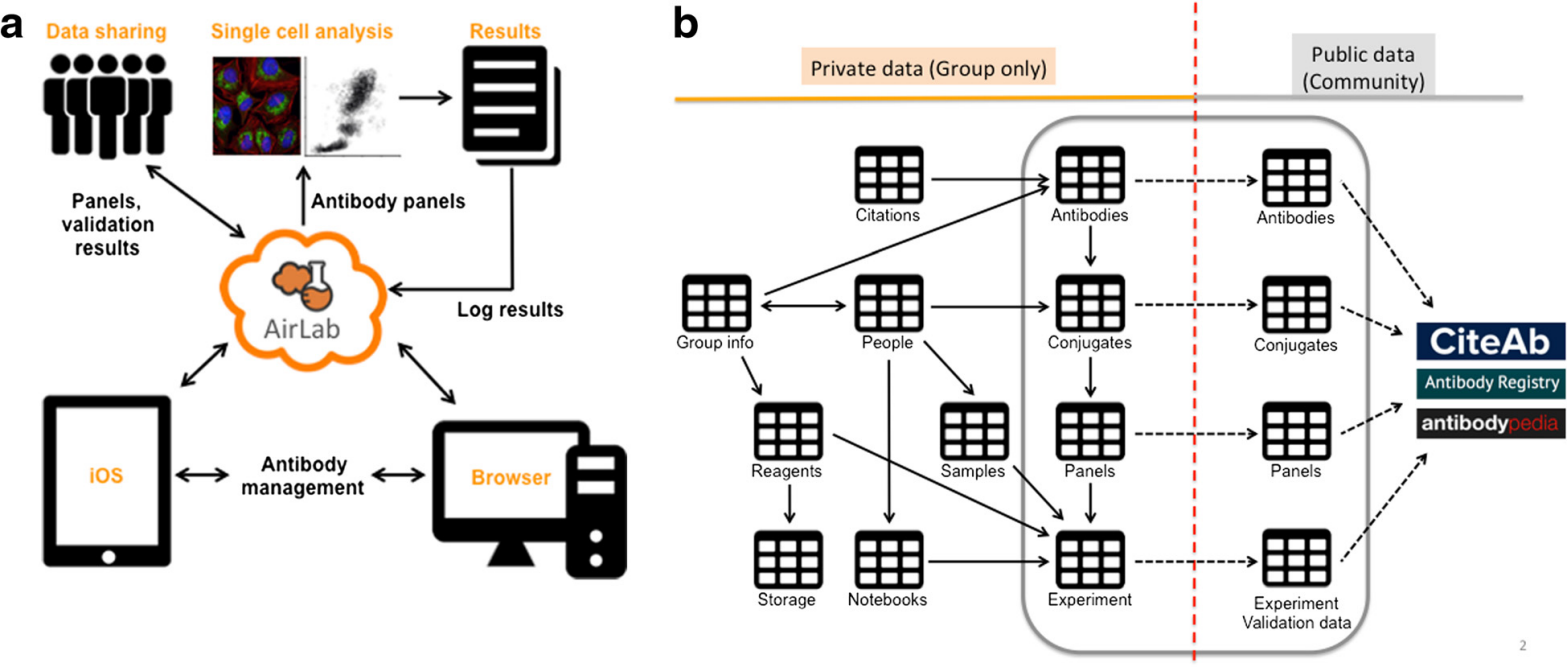
**a** Schematic of the AirLab platform. AirLab is a cloud-based tool with browser and iOS interfaces that powers antibody inventory, panel and experiment design and execution, and experimental data management. **b** AirLab also enables data sharing within a laboratory or with the community. The database schema summary depicts the main tables and relationships amongst them in the database. Data sharing is user-controlled

To address these needs, we have developed AirLab, a cloud-based tool to manage and share antibody-based single-cell research. AirLab was optimized for the maintenance of antibody inventories and antibody data sharing (Fig. 1a). AirLab streamlines the processes of purchase, vendor data acquisition, organization, storage, and antibody panel creation and enables validation data handling and sharing (Fig. 1b). Furthermore, AirLab can be customized for inventory of other entities in the laboratory, such as primers or patient samples, providing the basis for a full virtualization of the laboratory. Data are shareable among laboratory members and, in a user-controlled manner, with the community (Fig. 1). AirLab has both web browser and iOS app front-ends, bringing laboratory data into scientists’ pockets. We expect that this comprehensive tool, capable of handling multiple data types and laboratory entities, will save time and money for laboratories involved in large-scale antibody-based research and will ultimately develop into a database for sharing of antibody validation data.

## Methods

AirLab was written in PHP and employs a MySQL datastore. The PHP app can be deployed from a LAMP-stack-capable Linux server. The front-end web interface was written in HTML-Java script. AirLab is available upon registration and acceptance of terms and conditions at http://airlab.bodenmillerlab.org. At the time of publication of this article, AirLab was hosted in Google Cloud Services using SQL service and Google Storage Solution. Data are encrypted and all transactions are protected by user-specific tokens and passwords. The iOS application was written in Objective-C and Swift; the app can be downloaded from the App Store (www.appstore.com) for use with an account created at http://airlab.bodenmillerlab.org. The source code for the SQL database, and the web and iOS versions is available at http://www.bodenmillerlab.org/research-2/software-2/.

## Software features

### Registration and creation of accounts

A user must first create an individual account. After account creation, users are prompted to create a laboratory or research group account or to join an already existing group. Group creators are super administrators who can invite additional members and configure access rights. Invited users, if new to the platform, are prompted via email to accept the invitation and create an account. Group members have access to all group-level data and, based on rights granted by the administrator, can perform various tasks. Users also have private access to user-level data such as electronic laboratory notebooks.

### Inventory management

AirLab provides a simple way to manage all reagents in the laboratory (Fig. 1). Any user can place a reagent request, either by creating a new reagent in the database or by requesting from an existing record. Full support for reagent requests, including a convenient shop assistant in iOS, is implemented (Additional file 1: Figure S1a). Requested reagents are placed in a list. An authorized person (e.g., lab manager) must accept (Additional file 1: Figure S1a), modify, or reject the request. Upon acceptance of a request, items appear in the user’s *accepted* list. Accepted items should be marked as ordered when applicable by users with rights to do so (Additional file 1: Figure S1b), triggering a shift to the list of *ordered items*. Ordering occurs outside AirLab through the normal purchasing system of the institution. The price of the reagent can be input into AirLab for expense analysis if desired. Upon receipt, any laboratory member can mark the reagent as arrived (Additional file 1: Figure S1c). All actions, actors, and timestamps are recorded for fast examination of the purchasing process for every reagent (Additional file 1: Figure S1d, e). A quick response (QR) code that can be read with an iOS-device camera is generated for every reagent and can be used with the AirLab tool to perform further actions. The vendor barcodes can also be scanned and added to the database to enable reordering and other actions.

**Fig. 2:**
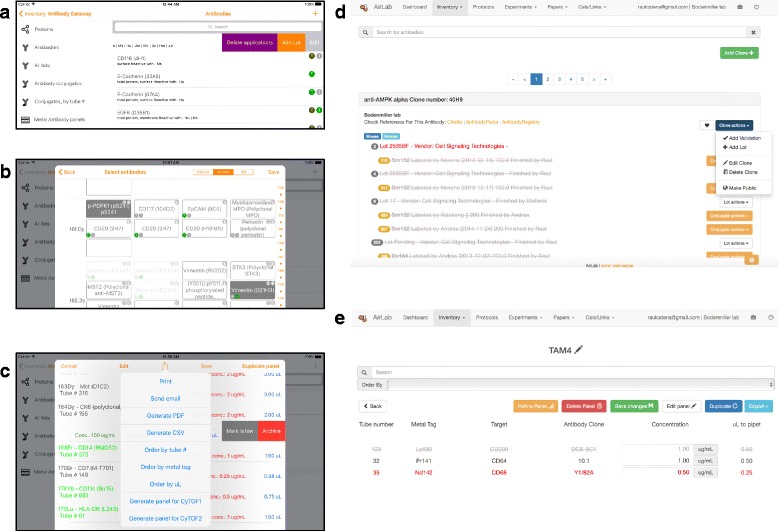
**a** Representative listing of antibody clones in the laboratory storage in the iOS tool inferface. Selection of a record uncovers options that can be performed over the record. **b** Panel building tool enables organization of antibodies per channel and offers basic information and detailed information (shown in iOS interface). **c** *Antibody Panel list* showing concentrations selected with calculations performed automatically based on stock concentrations (shown in iOS interface). This facilitates inventory: Those with little volume remaining are marked and those with no sample remaining are archived. Options for sharing and exporting, including as.conf files for control of CyTOF and CyTOF2 instruments, are shown. **d** Representative listing of antibody clones and associated lots and conjugates (shown in web tool interface). **e** *Antibody Panel list* showing concentrations selected with calculations performed automatically based on stock concentrations (shown in web tool interface)

If the antibody is ordered through the automatic shop assistant, the system will classify the reagent as an antibody. If the antibody purchase request is input manually, a switch must be selected to indicate that the reagent is an antibody (Additional file 1: Figure S2a). Antibodies are listed both in the *Reagent Inventory* section and in a section called *Antibody Gateway* (Fig. 2a; Additional file 1: Figure S2b). Specific information such as specificity, isotype, and epitope must be input (Additional file 1: Figure S2c). If the antibody has been requested through the automatic shop assistant, most of this information will be retrieved automatically from the vendor’s website; the user will be prompted to confirm the accuracy of the mined data (Additional file 1: Figure S2c).

Antibodies can also be input manually through the *Antibody Gateway*. When done in such a way, only an abstract antibody clone entity is created. An actual reagent tube (or lot number) must be added subsequently (Additional file 1: Figure S2d, e). Furthermore, antibodies conjugated with a fluorophore, adaptor epitopes like biotin, or a metal isotope can be added to the *Conjugates* list linked to a particular antibody clone entity (Additional file 1: Figure S2e, f) may be added. Upon conjugate creation, the system will suggest a number for the tube. We strongly suggest the use of this number. In this way, all information on a particular antibody is linked. In our laboratory, more than 900 conjugates are tracked using numbers automatically assigned.

### Samples and non-commercial reagents

Inventory of antibodies, samples, and other laboratory-generated reagents can be handled by AirLab (Fig. 1b). To add an item, the user presses the “+” button in the *Samples* section and selects the type of sample or reagent to add. Custom entities can be defined and reused as templates (Additional file 1: Figure S1f). Customized information such as volume, concentration, or photographs can be linked to items. A QR barcode is generated for every inventoried item; the OR code can be scanned by the iOS app when actions are performed on the item. AirLab enables tracking of aliquoted samples and custom fields can be created for aliquots (Additional file 1: Figure S1g). Custom actions are available after swiping over a registry (Additional file 1: Figure S1h), such as editing, aliquoting, marking as low, finished, or archived.

### Antibody panel creation

Panels of labeled antibodies can be created in AirLab (Fig. 1b). A *Panel Creation Tool* (Fig. 2b; Additional file 1: Figure S2g–i) allows visualization and selection of different conjugates, one per channel, to generate antibody panels of 40 antibodies or more. These panels can be edited, duplicated, and shared (Fig. 2c; Additional file 1: Figure S2g–i). Calculations for antibody preparation are done automatically after concentration input (Fig. 2c). Additionally, panels can be exported in several formats, including CSV, PDF, and.conf; the latter can be directly imported into CyTOF® software (Fig. 2c). Data can also be modified by the user during the experiment, for instance, allowing tubes to be marked as empty or low directly, which informs other laboratory members of the status of finished reagents instantaneously. *Panels* will display the AirLab assigned conjugate numbering system, which greatly facilitates the collection of samples from the inventory.

### Antibody validation data

Importantly, validation data can be linked to the antibodies. These data are very valuable and include optimal conditions, titration data, and validation results that aid users developing antibody panels (Additional file 1: Figure S3a). Information can be shared within the user’s laboratory or publically. Should the researcher want to make the data public across the scientific community (for instance, after successful publication of results in a journal), a simple click will enable this through a generated link (Fig. 1b). Use of the data sharing function in AirLab can drastically reduce the time spent by researchers who are performing antibody-based analyses. Further, AirLab connects directly with relevant online resources for antibody-based research such as antibodypedia [9] and the Antibody Registry (http://www.antibodyregistry.org), which act as interactive repositories for antibody information. Additionally, AirLab connects to CiteAb [10], an online database for antibody-based search of scientific article citations, which is currently the most reliable way of predicting the functionality of an antibody for a given application. Furthermore, AirLab is conceived as a crowdsourced repository, where researchers can add data and vote (thumbs up/down system) upon completion of validation experiments to indicate whether particular antibodies are specific and reproducible for a particular application such as flow cytometry or immunofluorescence, thus increasing the value of the tool.

### Storage management

AirLab also provides means to virtualize the laboratory and to specify the location of all samples and reagents (Additional file 1: Figure S4). Rooms, refrigerators, and freezers, liquid nitrogen tanks, racks, shelves, and boxes can be virtualized hierarchically using this tool. Reagents are linked to containers and information on storage sites (and amounts of samples remaining) can be accessed directly. In addition, labels can be generated and printed through AirLab to facilitate tracking.

### Electronic laboratory notebook

AirLab includes a simple but efficient electronic laboratory notebook (ELN). Text (which can also be dictated orally to iOS devices), images (from the hard drive or directly from the camera of the mobile device), well templates, and other files, such as Microsoft Word, Excel, and PowerPoint, and PDF files can be seamlessly included (Additional file 1: Figure S3a–d). Antibody panels defined in the *Antibody Gateway* section, other types of samples, or protocols can also be included in one click (Additional file 1: Figure S3e). ELNs can be shared with other laboratory members or with other researchers with an AirLab account. Use of the AirLab ELN limits the ability of users to manipulate data. An experiment’s creator can make modifications until sections of the notebook (such as text sections or images) are “closed”. Once a section is closed, it can no longer be modified (Additional file 1: Figure S3f). All members involved in a project can create new experiments and all can read all experimental descriptions; however, only the creator of an experiment is able to log data from an experiment, enforcing accountability. After publication of results in scientific journals, sections of ELNs can be set for public access.

AirLab is intended to virtualize all aspects of research and to consolidate all important data pieces (reagents, results, samples, protocols, scientific literature) in a single accessible format. Therefore, other functions are available or under development. A simple PubMed reference manager is included, as well as protocol builders and viewers and calculators. A general overview of the different types of registries that AirLab can handle and how they interact is depicted in Fig. 1b.

## Discussion

Research laboratories are complex environments where thousands of objects such as samples, reagents, instruments, storage solutions, people, laboratory notebooks, and digitized data files coexist. Multiple general databases and tools (such as Filemaker [11], Microsoft Office tools [12], Google Documents [13]) and dedicated research and laboratory software tools (such as Labguru (http://www.labguru.com), Quartzy (https://www.quartzy.com), and Endnote [14]) have been developed to aid inventory and information storage. Lacking, however, was a comprehensive software solution that handled the organization of antibodies (and other reagents) and associated data within a laboratory that could interface with publically available data. Additionally, in recent years powerful computers (like smart phones and tablets) have colonized nearly every pocket, but, despite their attractive features, these devices had not been integrated into research environments. We present here our development of a cloud platform that addresses in a global manner what other tools address only partially. The AirLab platform was engineered to work effectively with mobile devices and to interface seamlessly with internet sources. AirLab facilitates organization of data on large collections of antibody stocks and streamlines the processes of purchasing, storage, antibody panel creation, results logging, and antibody validation data sharing and distribution. Designed with antibody-based experiments in mind, the software can be easily adapted to tracking of any type of reagent and any type of high- or low-throughput experiment.

Our laboratory, as well as other testers in our university, use AirLab routinely for organizing antibody inventories and in design and analysis of high-throughput research based on mass cytometry, a state-of-the-art technology that allows for the measurement of 40 or more (with the potential to expand to 100) parameters in thousands of cells (or tissues, in the case of our recently developed imaging mass cytometry). Use of AirLab has decreased the time required to plan experiments drastically. Furthermore, our antibody waste is now negligible. Other important advantages include accountability for use of valuable reagents, expenditure tracking, and enhanced reproducibility. Importantly, AirLab facilitates data sharing and searching thanks to the cloud-based system. AirLab is a laboratory software solution for research in the digital age. The cloud-based platform in conjunction with mobile gadgets enables ready access to relevant information, reducing the logistical overhead of research that has become increasingly convoluted due to application of multiparametric experiments. A cloud-based system also represents a unique opportunity to aggregate data on antibody performance, which is invaluable for all antibody-based applications.

## Additional file

Additional file 1: Figure S1. Inventory management. **a** Requested reagent and buttons for laboratory managers to accept, reject, or modify a request. **b** Approved request. Managers can mark the reagent as ordered once the purchase order to the vendor is placed. **c** Purchased reagent. All members can mark reagents as arrived. **d** Information for purchased reagents showing units in stock. **e** Details on reagent unit purchase and storage. **f** Sample management system allows entry, management, and aliquoting of user-defined samples. **g** Sample information. **h** Contextual actions for sample registries. **Figure S2**. Antibody management tools. **a** Antibody purchasing tool, streamlines requests for antibody purchase. **b** Proteins list. Tapping shows list of clones available for that protein (see panel **d**). **c** Edit antibody clone tool collects basic information for an antibody registry. **d** All available clones, including specificities, sources, and applications. Contextual actions are available after swiping the row. **e** Conjugates list displays the conjugates per antibody lot. **f** Add conjugate window allows entry of conjugates by specifying the lot and tag. **g** Panels list. **h** Antibody panel building tool shows all conjugates; the user selects one conjugate per channel. Long tap displays available information for conjugate. **i** Antibody panel presentation mode allows users to set concentrations and record inventory information (finished, low) and export the panel as CSV or CyTOF templates. **Figure S3**. Electronic laboratory notebook (ELN). Simple but efficient. Users can insert common file types, **a** images, **b** Microsoft Office files, **c** custom plate templates, **d** photographs, and **e** antibody panels defined in the Antibody gateway section. **f** After entries are finished, sections can be locked to prevent future modification. **Figure S4**. Storage tools. Users can virtualize the laboratory by **a** room, **b** fridges, freezers, tanks, etc. **c** shelves and racks, or **d** boxes. **e** Functions available upon clicking (removing, relocating, and mark as finished). (PPTX 10226 kb)
