# Supplementary material for: AirLab: a cloud-based platform to manage and share antibody-based single-cell research
Source: Genome Biol. 2016 Jun 29;17:142. doi: 10.1186/s13059-016-1006-0 (PMC4928244; doi:10.1186/s13059-016-1006-0)
Supplement: Additional file 1: Figure S1. — Inventory management. a Requested reagent and buttons for laboratory managers to accept, reject, or modify a request. b Approved request. Managers can mark the reagent as ordered once the purchase order to the vendor is placed. c Purchased reagent. All members can mark reagents as arrived. d Information for purchased reagents showing units in stock. e Details on reagent unit purchase and storage. f Sample management system allows entry, management, and aliquoting of user-defined samples. g Sample information. h Contextual actions for sample registries. Figure S2. Antibody management tools. a Antibody purchasing tool, streamlines requests for antibody purchase. b Proteins list. Tapping shows list of clones available for that protein (see panel d). c Edit antibody clone tool collects basic information for an antibody registry. d All available clones, including specificities, sources, and applications. Contextual actions are available after swiping the row. e Conjugates list displays the conjugates per antibody lot. f Add conjugate window allows entry of conjugates by specifying the lot and tag. g Panels list. h Antibody panel building tool shows all conjugates; the user selects one conjugate per channel. Long tap displays available information for conjugate. i Antibody panel presentation mode allows users to set concentrations and record inventory information (finished, low) and export the panel as CSV or CyTOF templates. Figure S3. Electronic laboratory notebook (ELN). Simple but efficient. Users can insert common file types, a images, b Microsoft Office files, c custom plate templates, d photographs, and e antibody panels defined in the Antibody gateway section. f After entries are finished, sections can be locked to prevent future modification. Figure S4. Storage tools. Users can virtualize the laboratory by a room, b fridges, freezers, tanks, etc. c shelves and racks, or d boxes. e Functions available upon clicking (removing, relocating, and mark as finished). (PP [file 13059_2016_1006_MOESM1_ESM.pptx]

## Slide 1
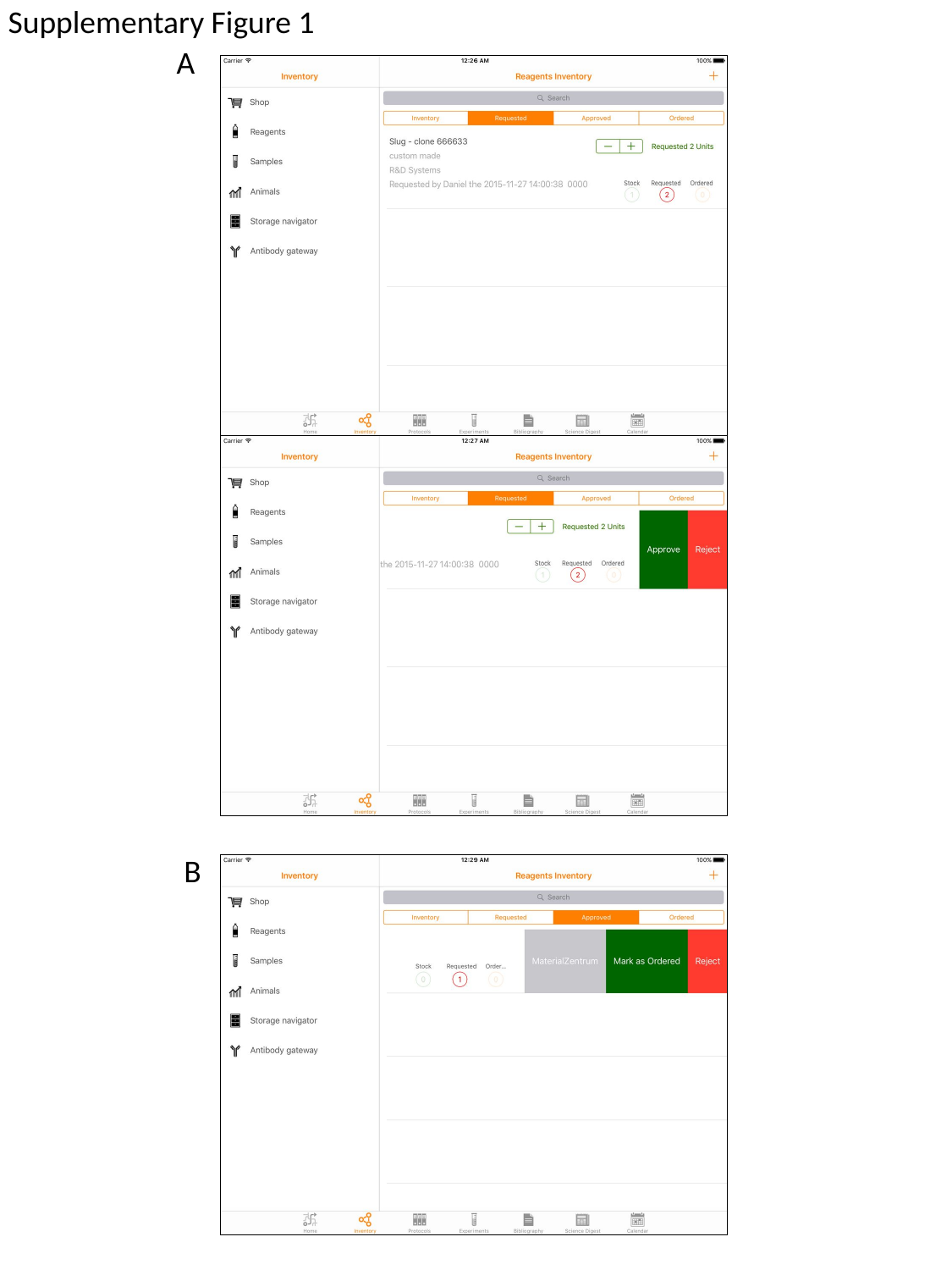

Supplementary Figure 1
A
B

## Slide 2
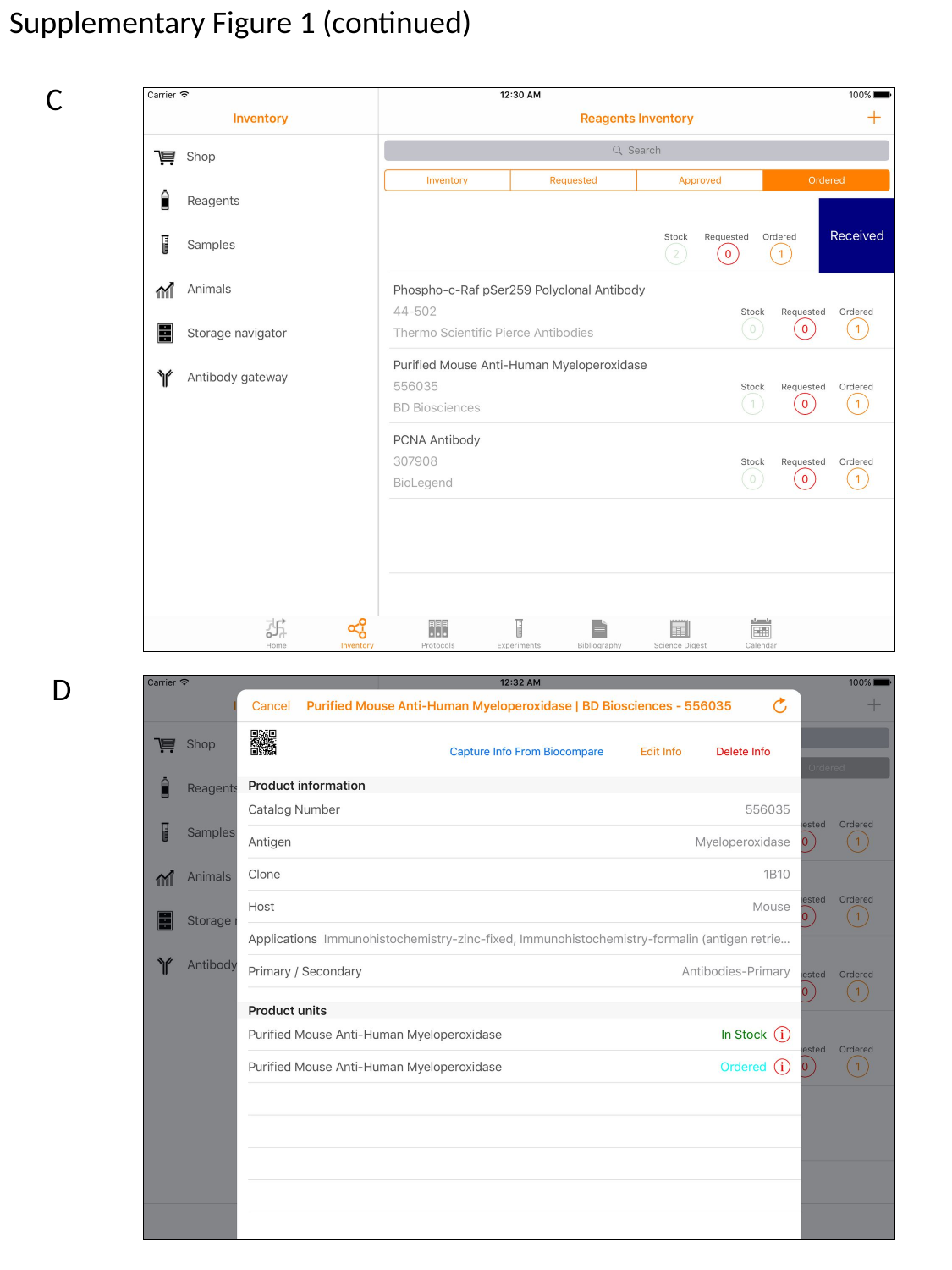

Supplementary Figure 1 (continued)
C
D

## Slide 3
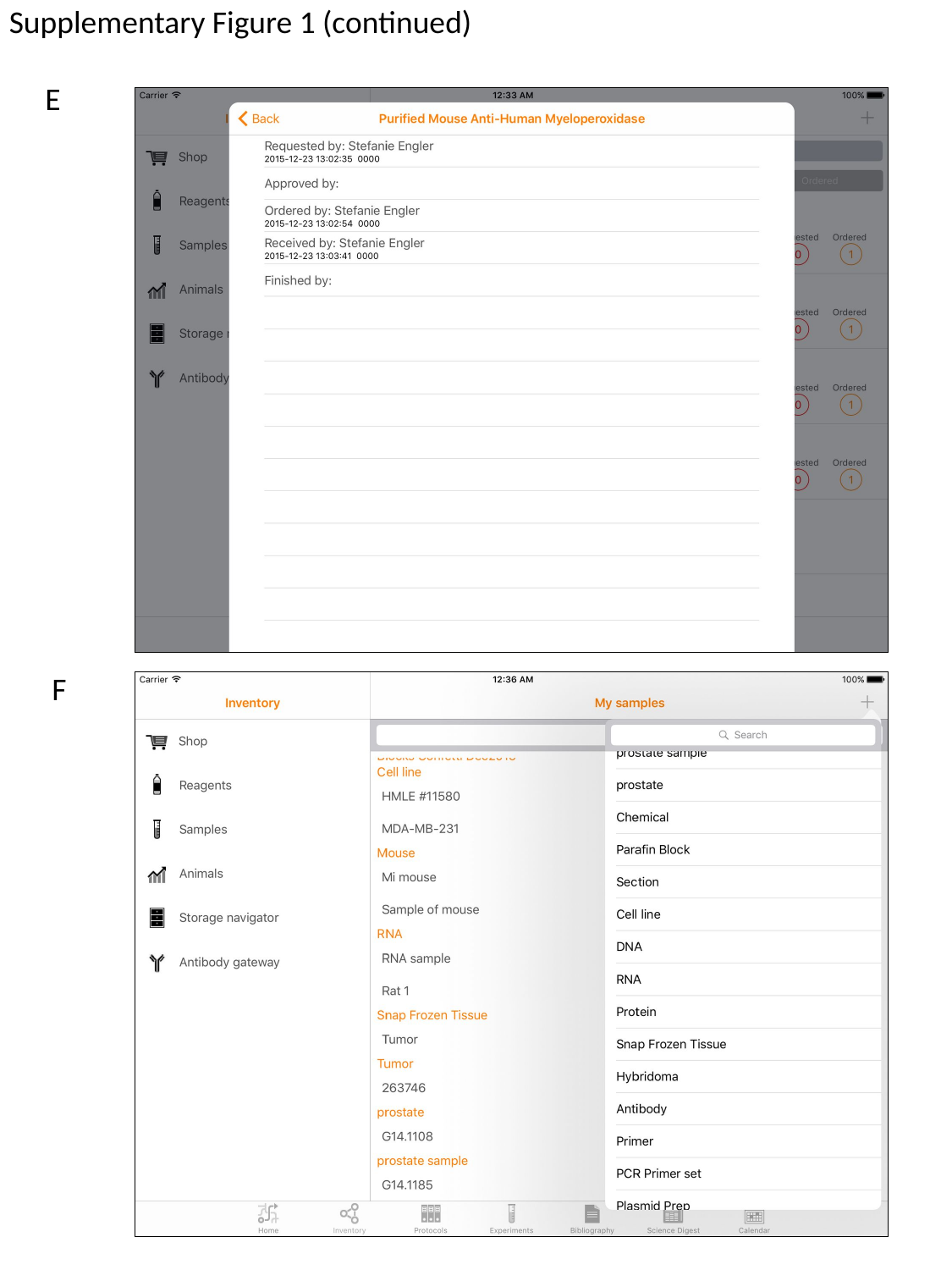

Supplementary Figure 1 (continued)
E
F

## Slide 4
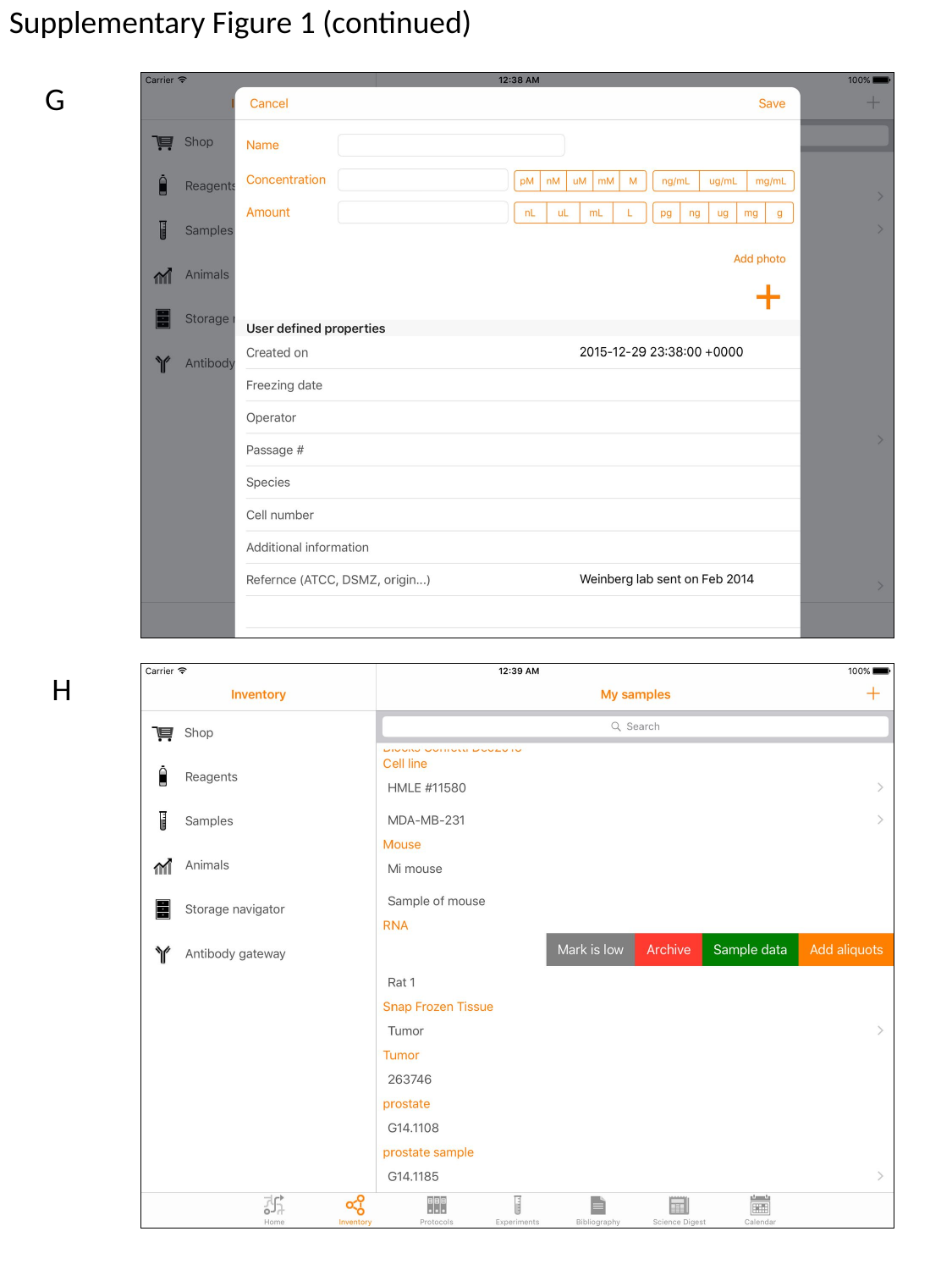

Supplementary Figure 1 (continued)
G
H

## Slide 5
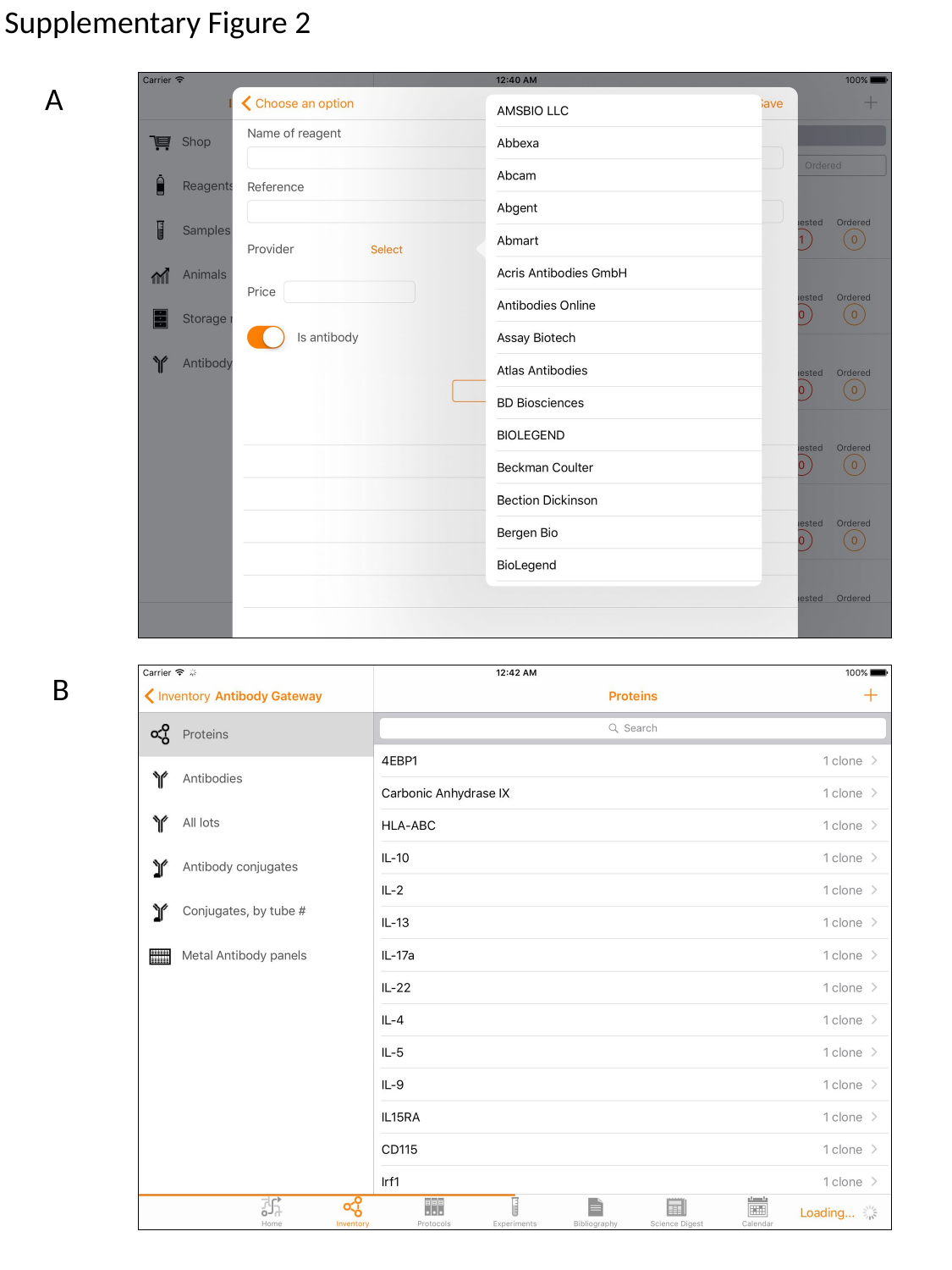

Supplementary Figure 2
A
B

## Slide 6
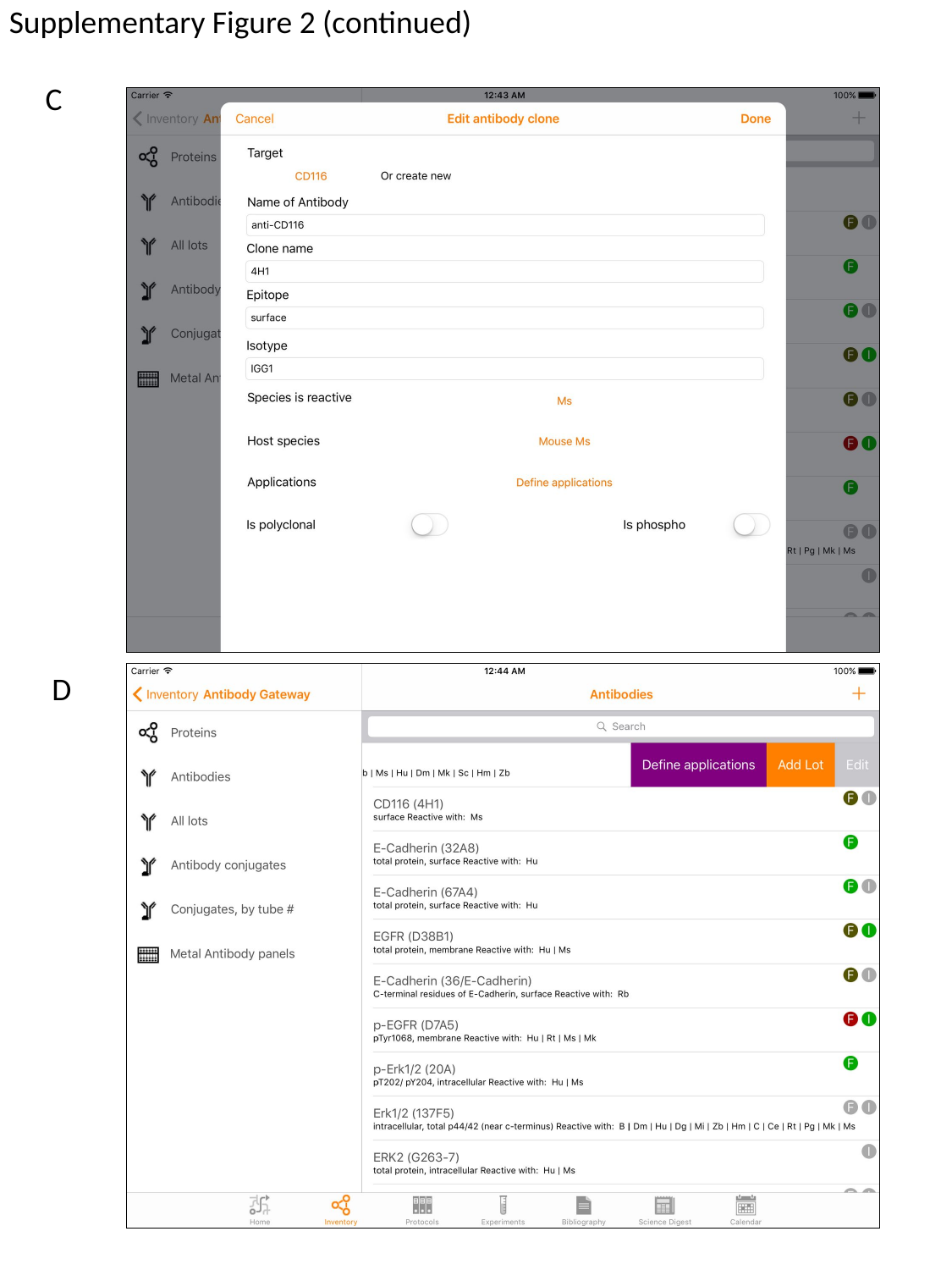

Supplementary Figure 2 (continued)
C
D

## Slide 7
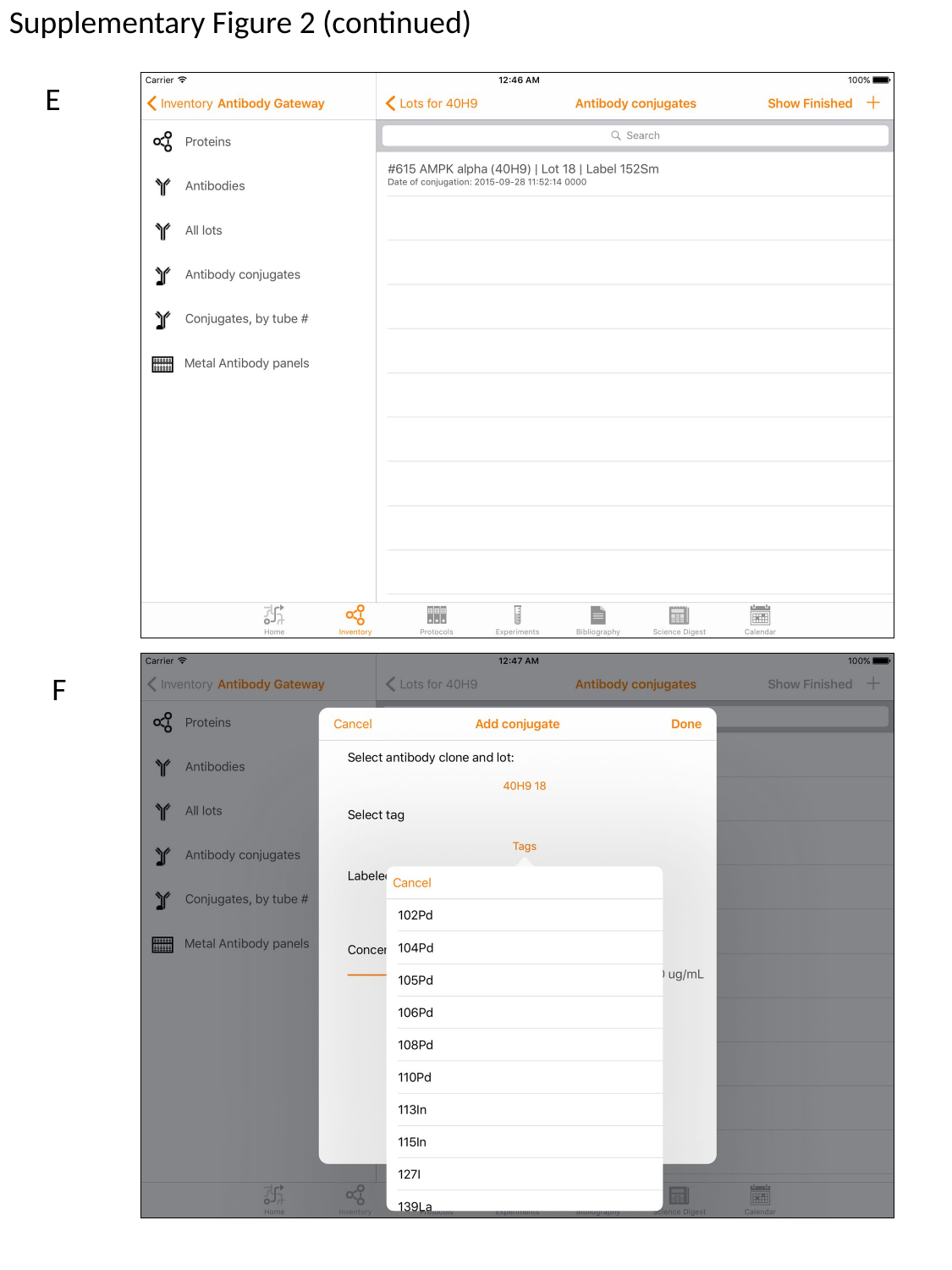

Supplementary Figure 2 (continued)
E
F

## Slide 8
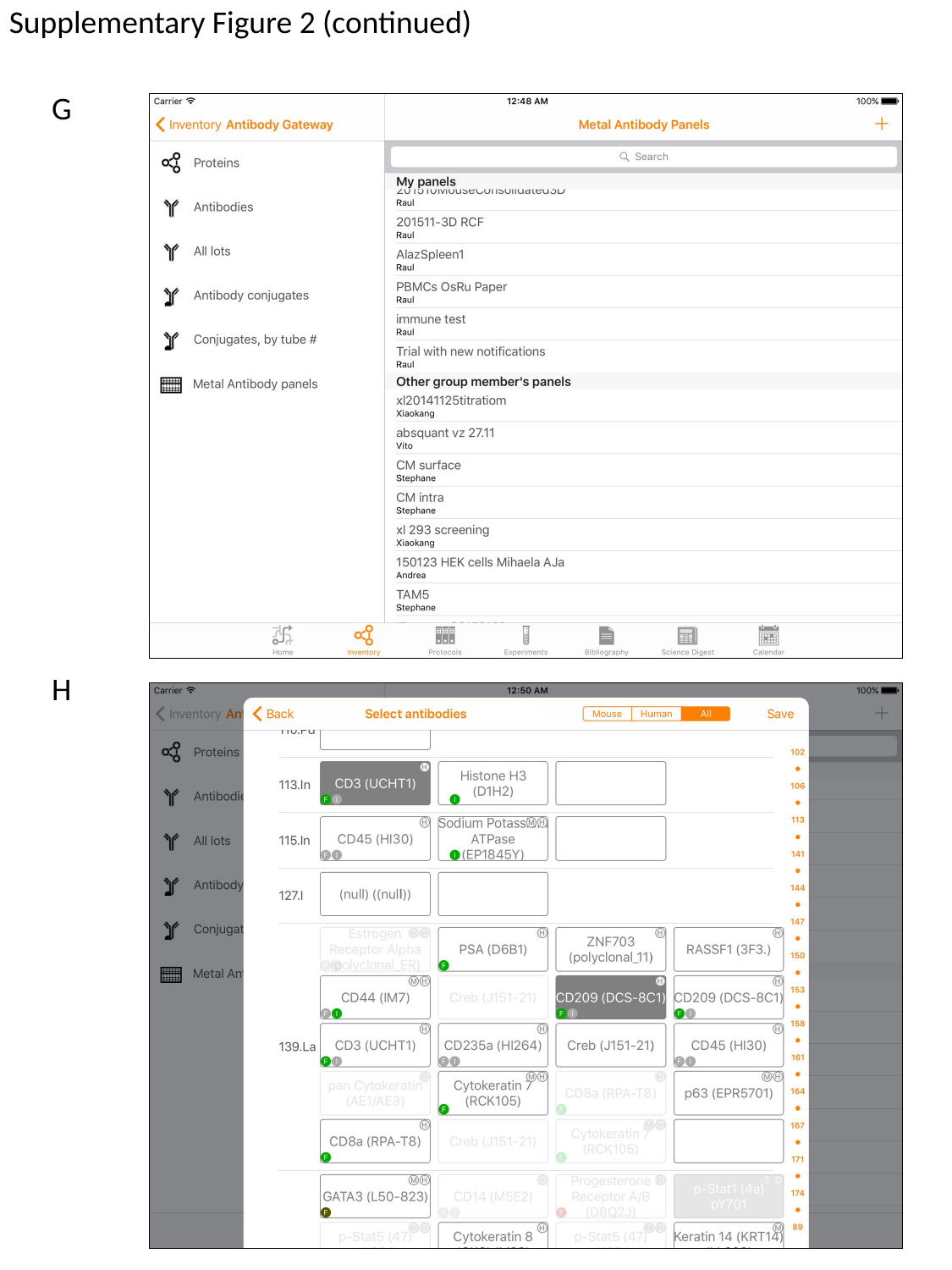

Supplementary Figure 2 (continued)
G
H

## Slide 9
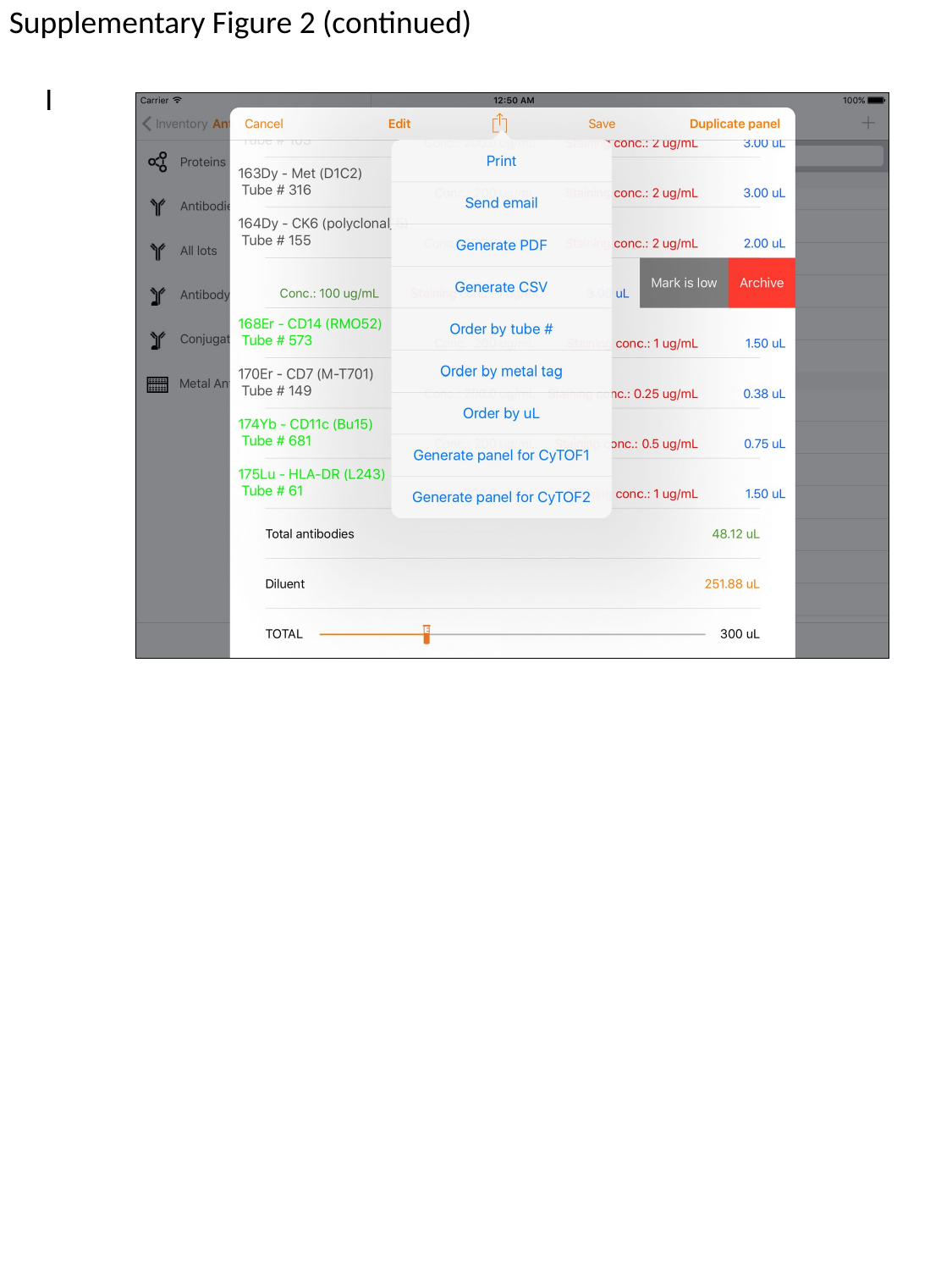

Supplementary Figure 2 (continued)
I

## Slide 10
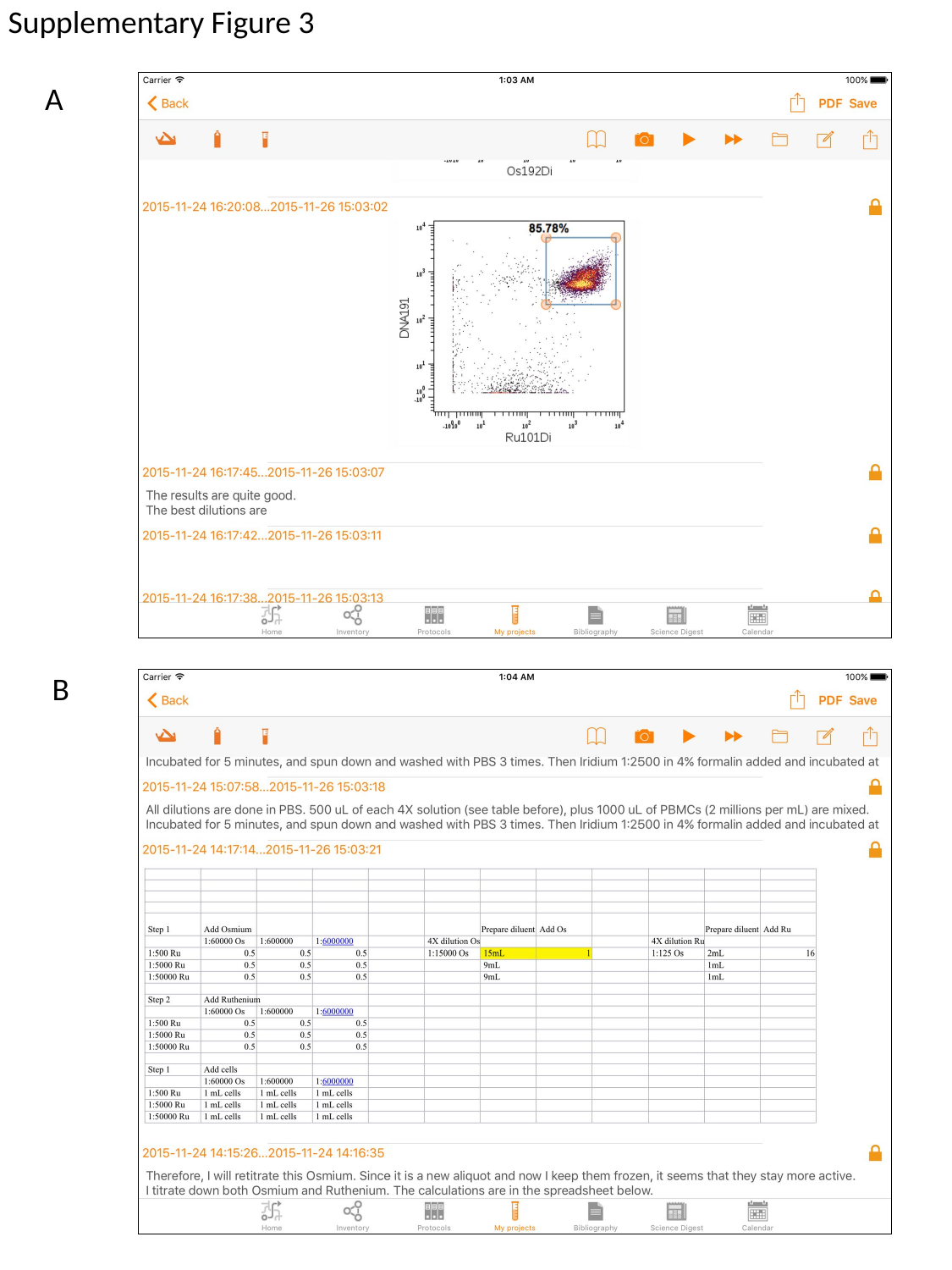

Supplementary Figure 3
A
B

## Slide 11
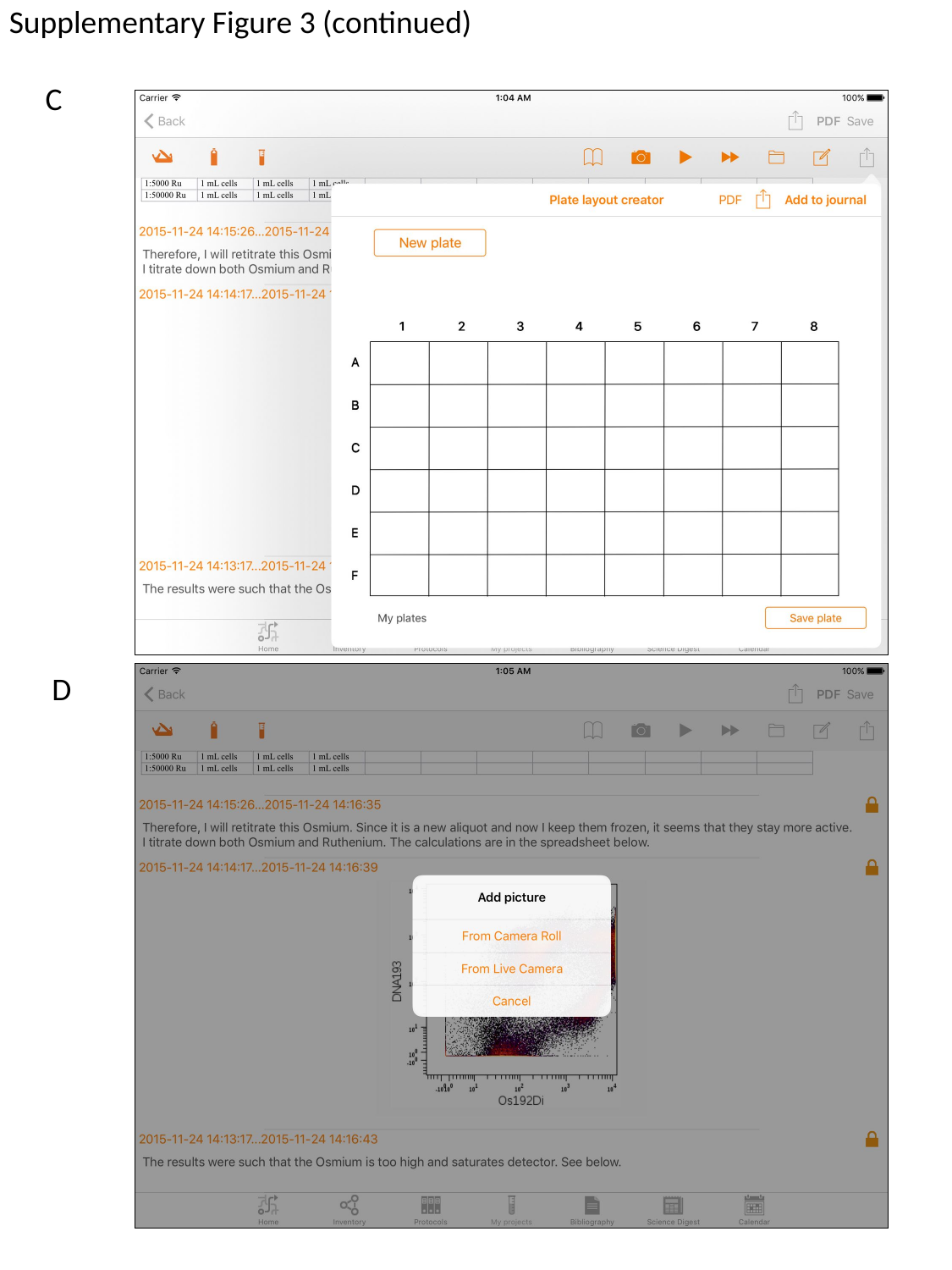

Supplementary Figure 3 (continued)
C
D

## Slide 12
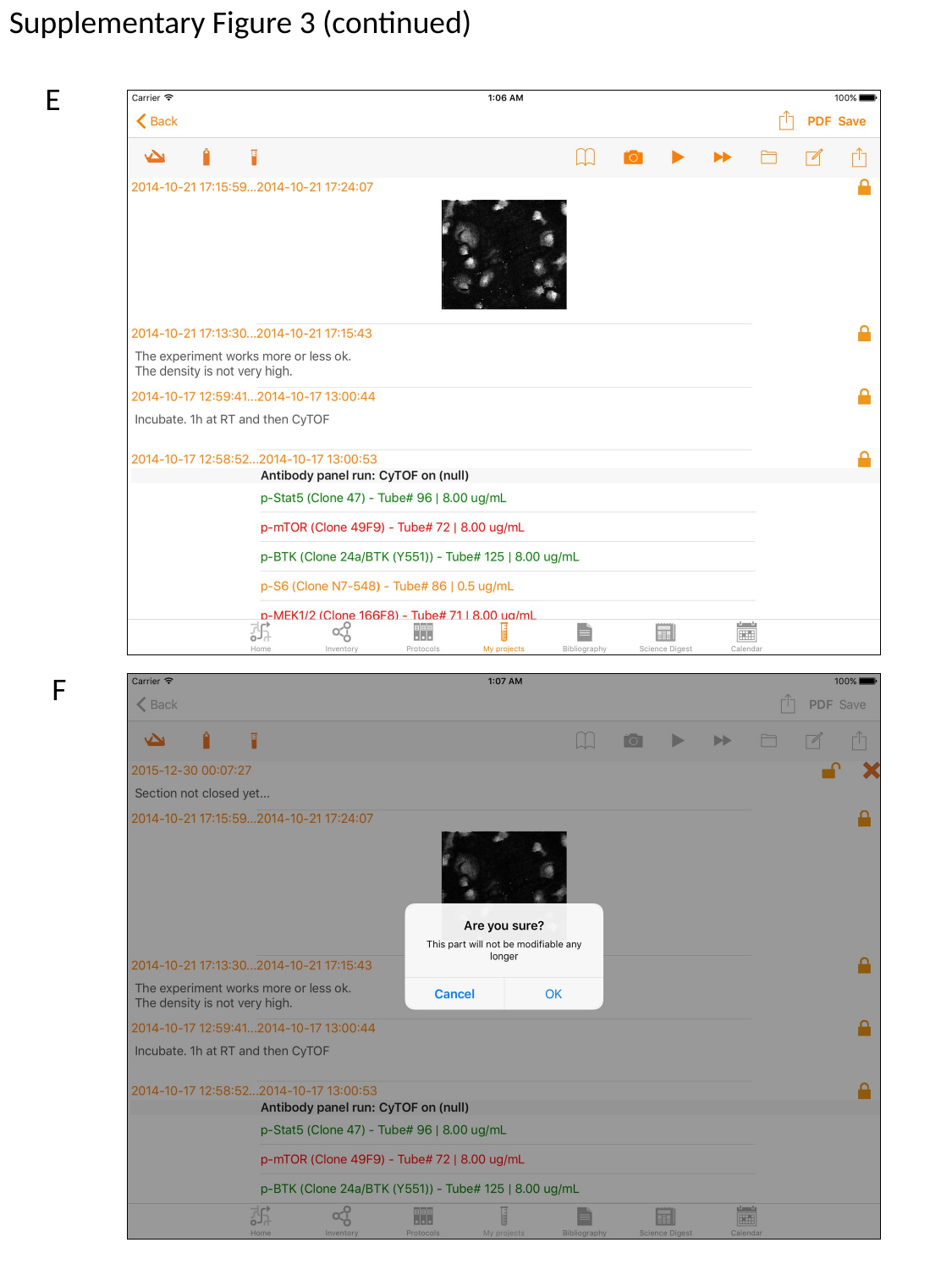

Supplementary Figure 3 (continued)
E
F

## Slide 13
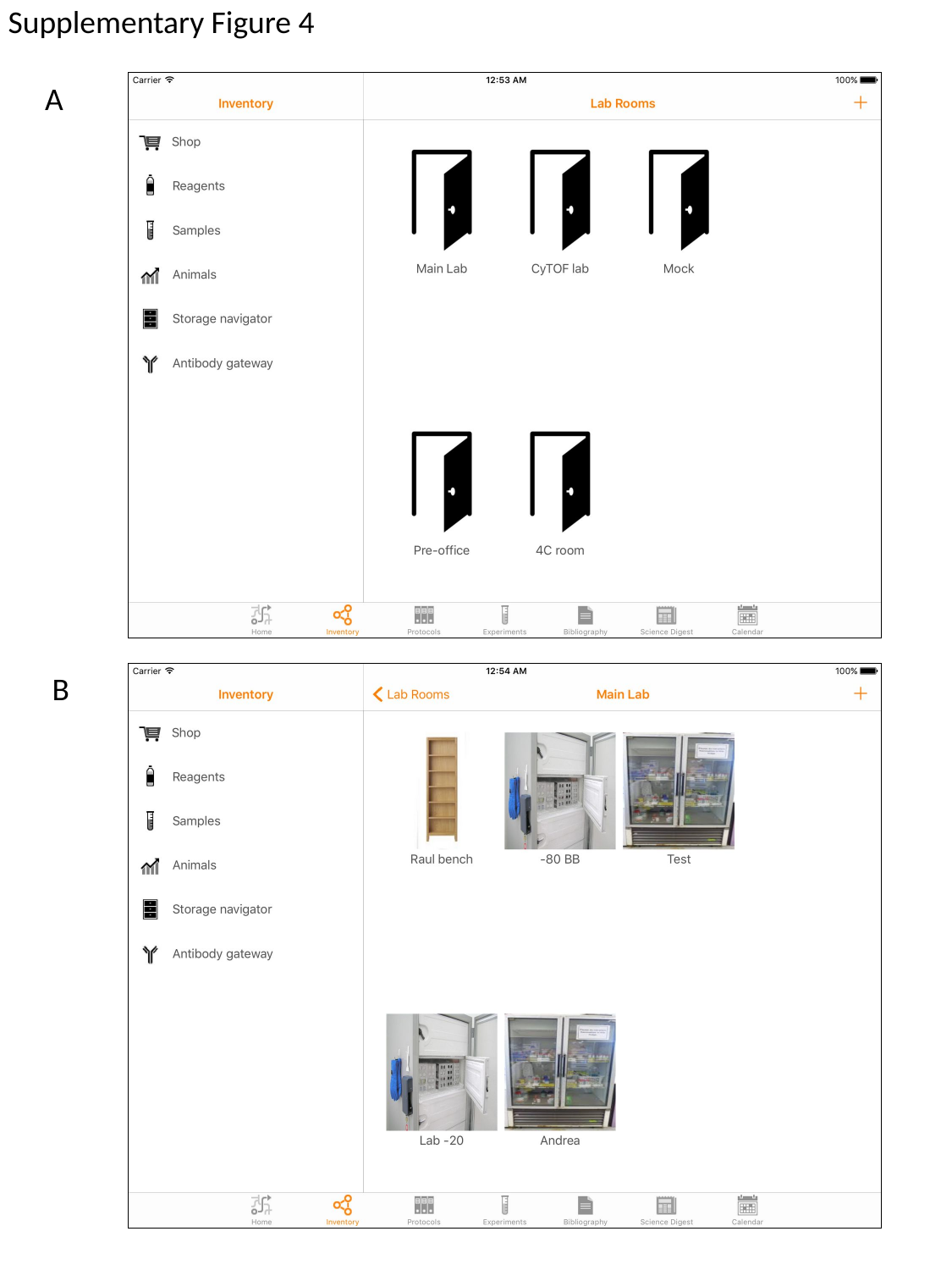

Supplementary Figure 4
A
B

## Slide 14
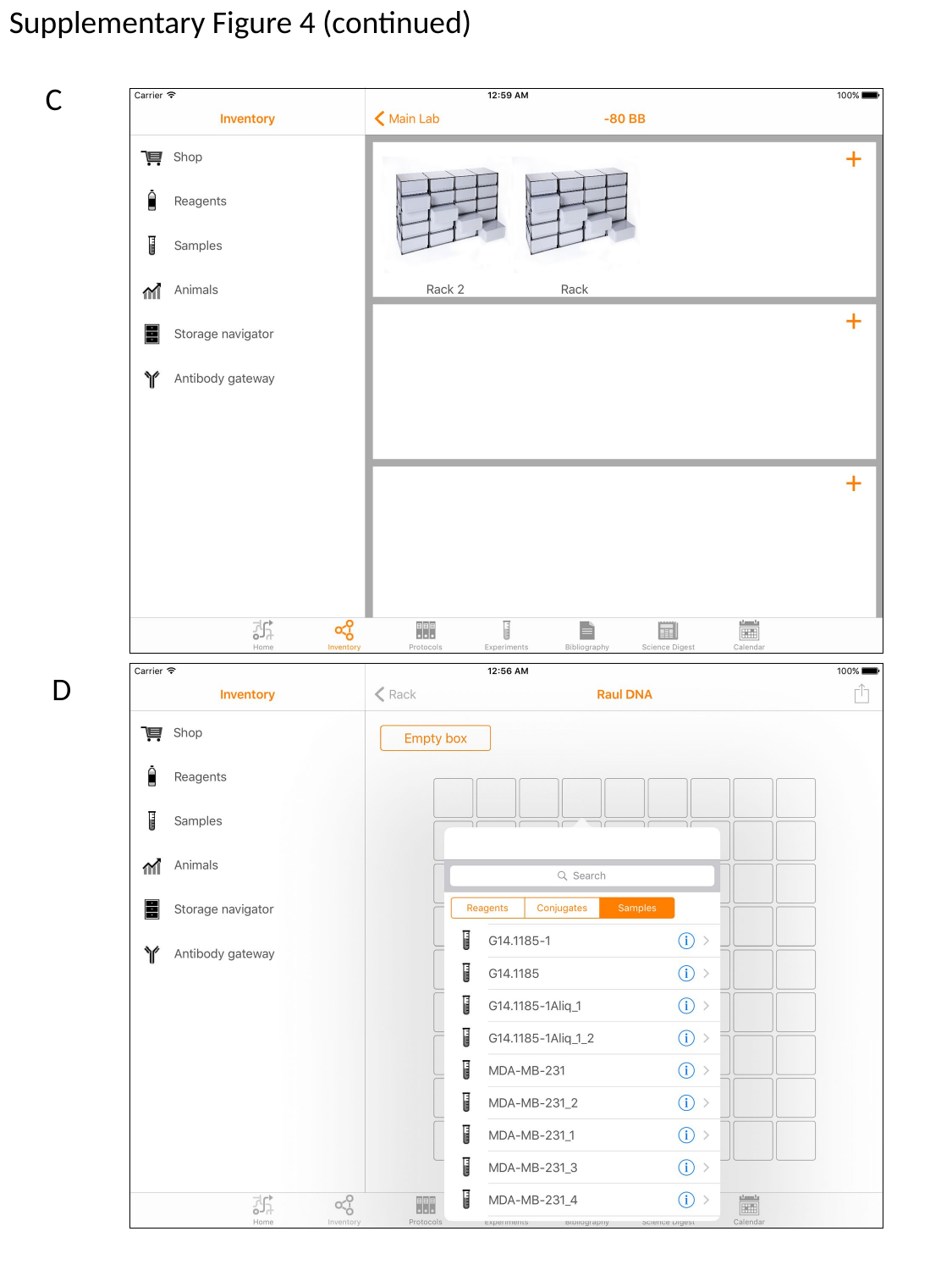

Supplementary Figure 4 (continued)
C
D

## Slide 15
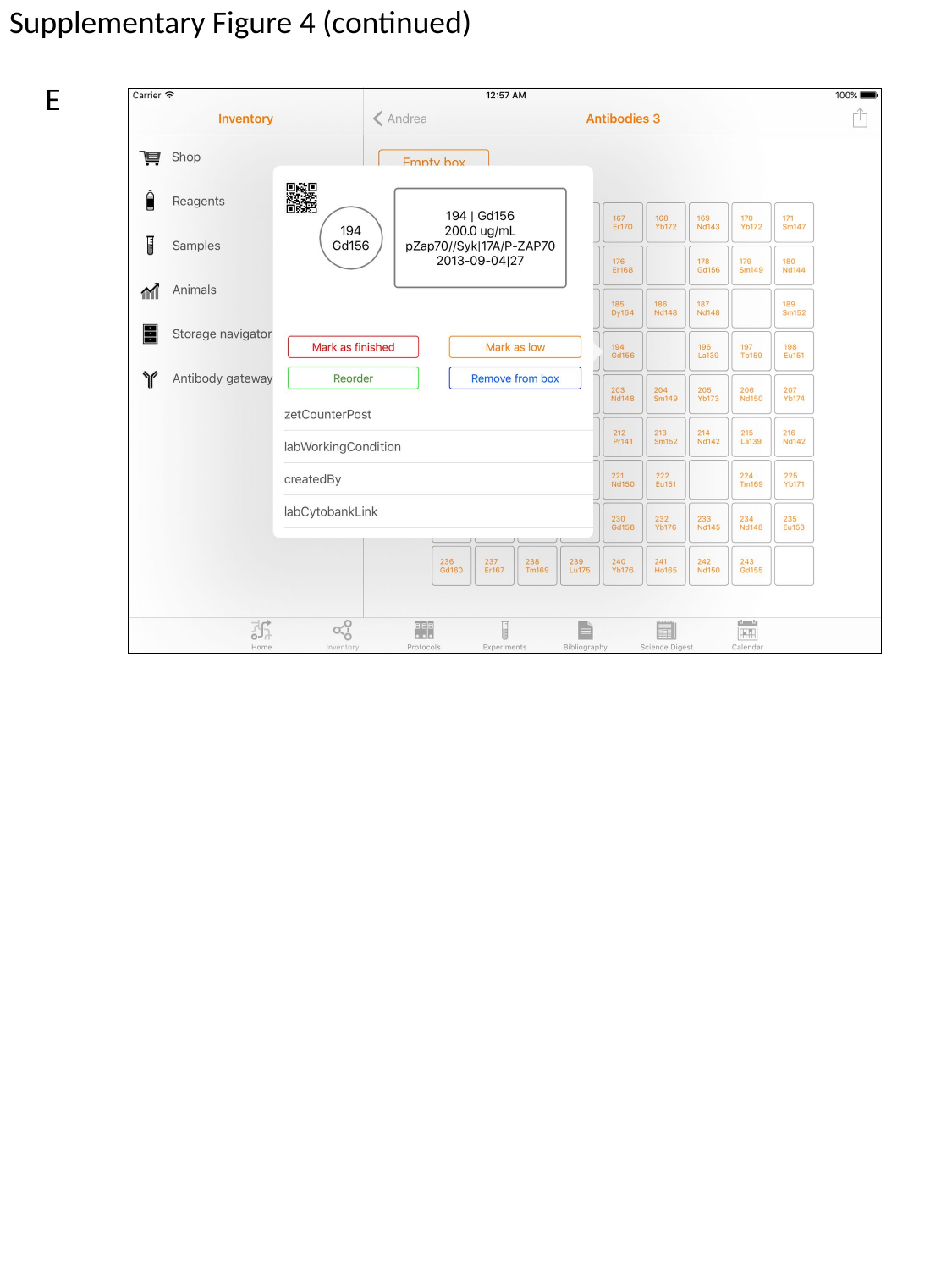

Supplementary Figure 4 (continued)
E
